# *Ex vivo *development, expansion and *in vivo *analysis of a novel lineage of dendritic cells from hematopoietic stem cells

**DOI:** 10.1186/1476-8518-8-8

**Published:** 2010-11-24

**Authors:** Shuhong Han, Yichen Wang, Bei Wang, Ekta Patel, Starlyn Okada, Li-Jun Yang, Jan S Moreb, Lung-Ji Chang

**Affiliations:** 1 Department of Molecular Genetics and Microbiology, University of Florida, Gainesville, Florida, 32610 USA; 2 Vectorite Biomedica Inc., Taipei, Taiwan; 3 Department of Pathology, Immunology and Laboratory Medicine, University of Florida, Gainesville, Florida, 32610 USA; 4 Department of Medicine, University of Florida, Gainesville, Florida, 32610 USA

## Abstract

Dendritic cells (DCs) play a key role in innate and adaptive immunity but the access to sufficient amount of DCs for basic and translational research has been limited.

We established a novel *ex vivo *system to develop and expand DCs from hematopoietic stem/progenitor cells (HPCs). Both human and mouse HPCs were expanded first in feeder culture supplemented with c-Kit ligand (KL, stem cell factor, steel factor or CD117 ligand), Flt3 ligand (fms-like tyrosine kinase 3, Flt3L, FL), thrombopoietin (TPO), IL-3, IL-6, and basic fibroblast growth factor (bFGF), and then in a second feeder culture ectopically expressing all above growth factors plus GM-CSF and IL-15.

In the dual culture system, CD34^+ ^HPCs differentiated toward DC progenitors (DCPs), which expanded more than five orders of magnitude. The DCPs showed myeloid DC surface phenotype with up-regulation of transcription factors PU.1 and Id2, and DC-related factors homeostatic chemokine ligand 17 (CCL17) and beta-chemokine receptor 6 (CCR6). Multiplex ELISA array and cDNA microarray analyses revealed that the DCPs shared some features of IL-4 and IL-15 DCs but displayed a pronounced proinflammatory phenotype. DCP-derived DCs showed antigen-uptake and immune activation functions analogous to that of the peripheral blood-derived DCs. Furthermore, bone marrow HPC-derived DCP vaccines of tumor-bearing mice suppressed tumor growth *in vivo*.

This novel approach of generating DCP-DCs, which are different from known IL-4 and IL-15 DCs, overcomes both quantitative and qualitative limitations in obtaining functional autologous DCs from a small number of HPCs with great translational potential.

## Background

Dendritic cells (DCs) initiate primary and memory immune responses as well as activate innate immunity and therefore, play a pivotal role in immunotherapy [1]. Accounting for only 0.02-0.2% of the total white blood cells, the number of DCs that can be isolated from peripheral blood is limited [2]. When cultured with supplement of GM-CSF and IL-4, PBMCs or CD14-selected monocytes generate DCs at about 50% of the starting cell number. Furthermore, patients with cancer or chronic infections often suffer from a compromised immune system with increased myeloid suppressor cells and dysfunctional DCs [3-9].

The developmental origin and tissue distribution of various lineages of human versus mouse DCs are still not well defined [10-15]. Transgenic mouse studies have reported several transcription factors implicated in regulating DC differentiation, which include zinc finger protein Ikaros, PU.1, *rel*B, the helix-loop-helix (HLH) transcription factor inhibitor of DNA binding or differentiation 2 (Id2), interferon regulatory factor (IRF) 4 and 8, the Ets-domain transcription factor Spi-B, and the Notch family of proteins [14,16]. In addition, growth factors such as Flt3L, KL, TPO, TNFα, GM-CSF, IL-3, IL-4, and IL-6 have been shown to promote development and maturation of DCs [17-20].

Growth factors such as KL and Flt3L appear to be strictly required for the generation of DC progenitors from HPCs in culture [21]. In the laboratory, GM-CSF and IL-4 are routinely used to generate DCs from adherent PBMCs, and GM-CSF and TNF-α can induce differentiation of HPCs into interstitial DCs and Langerhan's cells in 12-14 days [22]. GM-CSF and IL-15, on the other hand, drive DC differentiation from monocytes and bone marrow (BM) but the role of IL-15 in myeloid lineage development remains poorly understood [23,24]. IL-15 is a member of the γC receptor family of cytokines which is expressed by a variety of cell types important to the survival of fibroblasts, T cells and natural killer cells. IL-15 has been shown to promote the survival of mature DCs through an autocrine antiapoptotic mechanism [25,26], and IL-15-derived DCs are reported to display Langerhans cell-like features with strong T cell activation potential [23,24,27,28].

Although DCs can be derived from PBMCs, BM or embryonic stem cells, the source and the amount of these progenitor cells are restricted. While *ex vivo *DC development and expansion approaches have been attempted, only a moderate number of DCs can be generated with the most efficient system reporting about 94 fold expansion of DCs from BM cells [29,30]. The scarcity and the variability of the various DC subsets have significantly hindered fundamental studies of this important lineage of immune cells. Innovative strategies that can reproducibly generate a large amount of functional DCs from a limited number of progenitor/stem cells are urgently needed. Here we report a novel *ex vivo *culture system that combines expansion of HPCs and differentiation of a unique lineage of DC progenitors (DCPs). This system supports expansion and development of both human and mouse HPCs and DCs. The total number of DCs generated under this system reached more than five orders of magnitude in 30-40 days, and the *ex vivo *differentiated DCs displayed antigen capture, T cell activation and tumor suppression functions similar to that of the peripheral blood and BM-derived DCs. Thus, a large number of autologous HPCs and DCs can now be routinely generated from a small number of CD34^+ ^HPCs for the study of immune cell development with potential of translational applications.

## Methods

### Cells and mice

CD34^+ ^cells used in this study were purified from BM, mobilized peripheral blood (MPB) or cord blood (CB) using magnetic beads (Miltenyi Biotec) following the manufacturer's instructions or purchased from AllCell Inc. (San Mateo, CA), Cambrex (Baltimore, MD) and National Disease Research Interchange (Philadelphia, PA). Buffy coats of peripheral blood of healthy donors were purchased from Civitan Blood Center (Gainesville, FL). PBMCs were isolated from buffy coats of healthy donors or from blood of cancer patients with approval of the Institutional Review Board of University of Florida. B lymphoblastoid cell lines (BLCLs) were generated by transforming peripheral blood B lymphocytes with EBV as described previously [31]. The BLCLs were propagated in complete RPMI-1640 medium (Gibco, Grand Island, NY) supplemented with 2 mM L-glutamine, 100 ug/ml streptomycin, 100 IU/ml penicillin and 10% heat inactivated fetal bovine serum (FBS) at 37°C with 5% CO_2_. The mouse fetal stromal cells were cultured in Minimal Essential Medium Alpha (Gibco, Grand Island, NY) supplemented with 2 mM L-glutamine, 100 ug/ml streptomycin, 100 IU/ml penicillin and 20% heat inactivated FBS (Gibco) at 37°C with 5% CO_2_. CT26 mouse colon carcinoma cell line was purchased from ATCC (catalogue no. CRL-2638) and cultured in Dulbecco Modified Eagle's Medium (Gibco, Grand Island, NY) supplemented with 2 mM L-glutamine, 100 ug/ml streptomycin, 100 IU/ml penicillin and 10% heat inactivated FBS (Gibco) at 37°C with 5% CO_2_. BALB/c mice were obtained from Jackson Laboratory (Bar Harbor, ME) with approval from the Institutional Animal Care and Use Committee of University of Florida.

### Antibodies and reagents

Fluorescein isothiocyanate (FITC)-conjugated Abs to CD4, CD8, CD11c, CD33, CD34, CD38, CD86, IFN-γ, and HLA-DR, phycoerythrin (PE)-conjugated Abs to CD4, CD8, CD11c, CD34, CD83, CD90, CD123, HLA-DR, IFN-γ, and TNFα, PerCP Cy5.5-conjugated Ab to CD8, CD33, PE-Cy7-conjugated antibodies to CD8, CD11b, CD11c, CD34, CD62L, CD40 and CD80, and allophycocyanin (APC)-conjugated Abs to CD1a, CD3, CD11c, CD14, CD33, CD69, CD83, CD90, CD133, IFN-γ and TNF-α were purchased from BD Pharmingen (San Diego, CA), eBioscience (San Diego, CA), Miltenyi Biotec (Auburn, CA), and Caltag Laboratories (Invitrogen, Carlsbad, CA) as listed in Additional file 1, Table S1. Isotype-matched antibodies were included as controls. HLA-A2 restricted, EBV BMLF1 GLC-peptide (amino acid 280-288, GLCTLVAML) pentamer was purchased from Proimmune (Springfield, VA).

### Lentivector preparation and gene transfer

Lentivectors (LVs) were constructed as described previously [32-34]. The growth factor cDNAs were amplified by PCR using primers designed to contain an optimized translation initiation sequence (-CCACC-5' to the initiation codon). The primers used in this study are listed in Additional file 2, Table S2. The amplified cDNAs were cloned into the self-inactivating pTYF plasmid behind the EF1α promoter. To generate feeder cells, mouse fetal stromal cells were multiply transduced with LVs at 10-50 infectious unit/cell in 12-well plates in a minimal volume of 0.3 ml per well. After 2 h, 0.5 ml of fresh media was added and cells were incubated at 37°C overnight. The infected cells were continuously propagated for more than 50 passages and stable lentiviral transgene expression was confirmed. The mouse CT26 tumor cells and DCs were transduced with LVs encoding a codon-optimized human HPV E6-E7 fusion protein and the chaperone protein calnexin as previously described [35].

### RNA extraction, RT-PCR and microarray analysis

RNA was extracted using Tri-reagent (MRC Inc., Cincinnati, OH) and oligo(dT)_15_-primed cDNA was made with MMLV reverse transcriptase (Promega Inc., Madison, WI). For semi-quantitative PCR, all reactions used the same serially diluted cDNA normalized to the mouse GAPDH (mGAPDH). The PCR amplification conditions were as follows: denaturing temperature, 95°C; annealing temperature, 55-62°C; extension temperature, 72°C; the amplification cycles were 25-35 cycles. Products were resolved by agarose gel electrophoresis and visualized by ethidium bromide staining. The PCR primers used in this study are listed in Additional file 2, Table S2.

For gene expression microarray analysis, RNA samples were harvested from purified CD34^+^HPCs, *ex vivo *cultured DCPs, and adherent PBMC-derived IL-4 and IL-15 DCs. These RNA samples were analyzed using Illumina Human RefSeq-8 Expression BeadChips. RNA quantity was determined with the Agilent RNA 6000 Nano Kit and Bioanalyzer. All samples displayed 28 S and 5.8 S peaks indicating intact full length RNA. Synthesis of double-stranded cDNA and *in vitro *transcription were performed with the Ambion Illumina TotalPrep kit according to manufacturers' instructions. For each sample, input quantity for the first strand synthesis was normalized to 200 ng. After *in vitro *transcription reaction, yield of purified cRNA was assessed with the RiboGreen assay and quality was assessed with the Agilent Bioanalyzer. BeadChip hybridization, staining and scanning were performed according to Illumina whole genome expression for BeadStation. For each sample, input of cRNA was normalized to 1500 ng. As control, Stratagene Universal Human Reference (SUHR) RNA was labeled with the Ambion TotalPrep kit. The labeled cRNA was used as interchip hybridization replicates and showed strong correlation. Biological replicate pairs were analyzed and for unnormalized data, the linear r^2 ^was greater than 0.94 for all replicates.

### Generation of mature DCs and antigen-specific immune cells

PBMCs were isolated after Ficoll-Hypaque density centrifugation (Sigma Aldrich, St Louis, MO). After plastic adherence, the adherent cells were cultured in 50 ng/ml GM-CSF and 25 ng/ml IL-4 (eBiosource International, Inc. Camarillo, CA) in serum-free AIM-V medium (Invitrogen, San Diego, CA) to generate immature DCs. Immature DCs were transduced with LVs, and treated with LPS (1 ug/ml) and TNFα (20 u/ml) for 24 hr to induce maturation. The mature DCs were loaded with 5 ug/ml of specific peptides. The non-adherent PBMCs were cocultured with irradiated (10 Gy) mature DCs, at a 20:1 ratio, in AIM-V supplemented with IL-2 (12.5 U/ml) and IL-7 (10 ng/ml) in 24-well plates. At day 12 of coculture, the T cells were restimulated or harvested for analysis as previously described [36,37]. The DCs of BALB/c mice were generated from BM of tumor-bearing mice and transduced with LVs as previously described [38].

### Quantitative cytokine and chemokine multiplexed enzyme-linked immunosorbent assay (ELISA) arrays

DCs were washed twice with PBS and cultured in AIM-V without growth factors and other supplements at a density of 10^6 ^cells/ml for 24 hr. The supernatants were collected and delivered to Quansys Biosciences (Logan, UT) for custom multiplexed sample testing as previously described [35]. Each sample was tested in triplicate. The list of cytokines and chemokines tested included: IL-1a, IL-1b, IL-2, IL-4, IL-5, IL-6, IL-8, IL-10, IL-12p70, IL-13, IL-17, IL-23, IFN-γ, TNF-α, TNF-β, Eotoxin, growth-related oncogene-alpha (GRO-α), monocyte chemotactic protein 1 (MCP-1), MCP-2, regulated upon activation, normal T-cell expressed and secreted cytokine (RANTES), I-309 and thymus and activation-regulated chemokine (TARC).

### Antibody and pentamer staining and flow cytometry

For antibody (Ab) staining, single-cells were suspended in PBS containing 2% FBS and 0.05% sodium azide and pre-incubated with anti-CD16/CD32 Ab for 10 min to block FcRs. Expression of cell surface markers was analyzed by standard four-color staining using FITC-, PE-, PE-Cy7 or PerCP and APC-conjugated primary Abs. To evaluate the expression of intracellular molecules, cells were washed and restimulated for 5 hr in the presence of brefeldin A (1 ug/ml) during the last 2.5 hr of culture. The stimulated cells were stained with anti-surface marker Abs, washed and permeabilized with the CytoFix-Cytoperm kit (BD Pharmingen), according to the manufacturer's instruction, then stained with anti-intracellular marker Abs, and analyzed by flow cytometry. For multimer staining, the resting T cells were stained with PE-labelled pentamer (Proimmune) for 12 min at room temperature, followed by FITC-labeled or PE Cy7-labelled anti-CD8 antibody for 30 min on ice and analyzed by flow cytometry. Data acquisition and analysis were done on a FACSCalibur and FACSAria using CellQuest and FACSDiva software, respectively (BD Biosciences, San Jose, CA), or Flowjo software (Tree Star, Inc. Ashland, OR).

### Analysis of antigen uptake

Immature DCs were harvested and washed with AIM-V twice and re-suspended in AIM-V at a concentration of 5x10^5 ^cells per ml. DCs were incubated with Dextran-FITC or OVA-FITC (Molecular Probes, Inc., Eugene, OR) at 37°C for 1 h; a parallel control was incubated at 4°C for 1 h. Cells were washed three times with cold FACS buffer, resuspended in 100 ul of cold FACS buffer, stained with APC-conjugated anti-CD11c Ab (BD Biosciences, CA) and analyzed by flow cytometry.

### *In vivo *DC vaccine tumor model

BALB/c CT26 colon cancer cells were transduced with LV-optE6E7 encoding a fusion protein of HPV16 E6/E7 to generate the CT26-E6E7 cell line. The BALB/c mice were inoculated with 1x10^5 ^CT26-E6E7 tumor cells subcutaneously. Seven days later the mice were vaccinated with 2-5 x10^5 ^immature DC/LV-optE6E7 or DC/LV-optE6E7/LV-calnexin derived from tumor-bearing mice, weekly for 2 weeks (n = 5 per group). Tumor size was measured over time using calipers and mean tumor volume (in mm^3^) was determined.

### Statistical analysis

The statistical analysis was performed using Student's t-test and GRAPHPAD PRISM 4 software.

## Results

### Expansion of CD34^+ ^HPCs and development of DC progenitors (DCPs)

**Figure 1:**
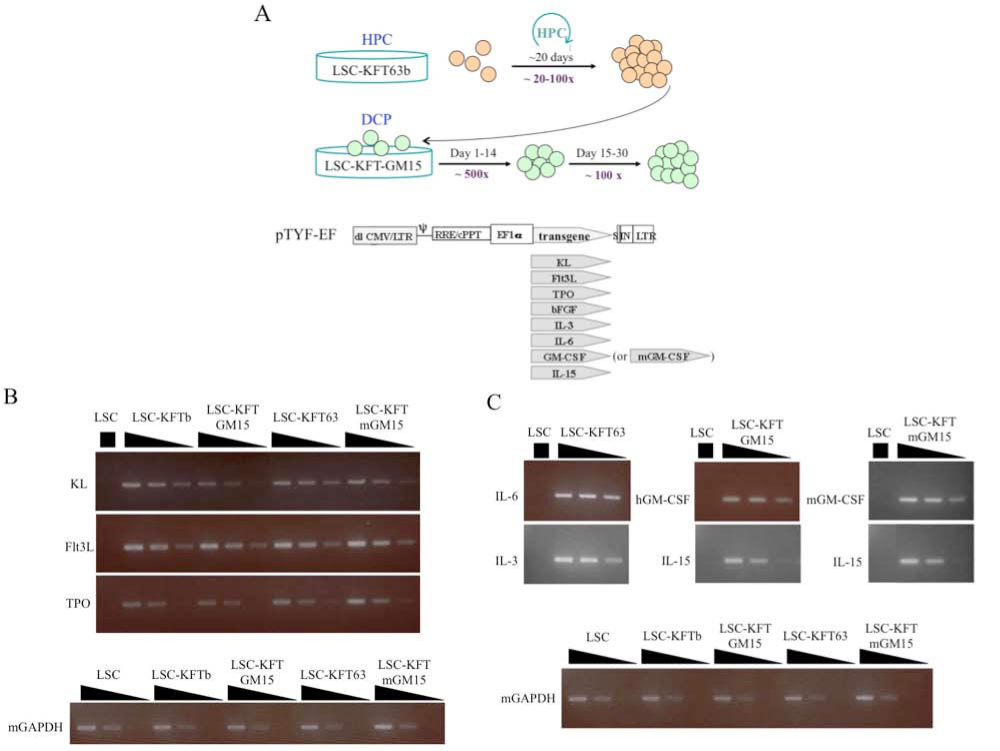
***Ex vivo *HPC to DC expansion and development system**. *(A) *Schematic representation of the LSC culture system and the lentivector constructs. The two LSC lines, LSC-KFT63b and LSC-KFT-GM15, produce the specified hematopoietic growth factors, which support the expansion of HPCs and DCPs. *(B) *and *(C) *Semi-quantitative RT-PCR analyses of lentiviral transgene expression in the LSC lines. All of the growth factor genes are human origin except for the mouse GM-CSF (mGM-CSF); the control endogenous mouse GAPDH (mGAPDH) gene expression is shown at bottom.

HPCs can expand in culture but have limited potential of maintaining hematopoietic stem cell phenotype and function [39]. We established a series of lentivector-modified stromal cell (LSC) lines to provide cell-free and cell-associated signals that can support continuous expansion of HPCs and differentiation of DCs (Fig. 1). The LSC lines include LSC-KFT (KL, Flt3L, TPO), LSC-KFTb (KL, Flt3L, TPO and bFGF), LSC-KFT63 (KL, Flt3L, TPO, IL-6, and IL-3) and LSC-KFT63b (KT, Flt3L, TPO, bFGF, IL-6, IL-3 and bFGF) for the expansion of HPCs, and LSC-KFT-GM15 (KL, Flt3L, TPO, GM-CSF and IL-15) and LSC-KFT-mGM15 (KL, Flt3L, TPO, moues GM-CSF, and IL-15) for the differentiation and expansion of human and mouse DCs, respectively. LSC-KFT, LSC-KFTb, LSC-KFT63 and LSC-KFT63b supported HPC expansion to similar extents, and the total expansion fold varied with individual donors. Under this culture condition, HPCs consistently expanded twenty to one hundred-fold in twenty days, followed by more than one thousand-fold expansion and differentiation into DCPs in thirty days (Fig. 1A). This dual culture system supports expansion and development of DCs from both human and mouse HPCs.

**Figure 2:**
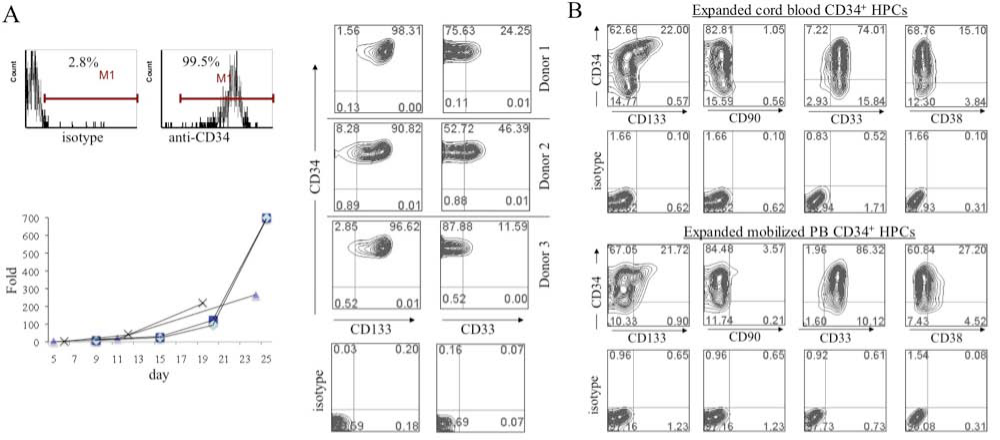
**Expansion of CD34+ HPCs and phenotype analysis**. *(A) Ex vivo *expansion kinetics of CD34+ HPCs in LSC culture. HPCs were purified using anti-CD34 Ab magnetic beads and the expansion kinetics on LSC-KFT63b of HPCs from four donors are plotted at bottom. The purified CD34+ cells were analyzed with anti-CD34, anti-CD133, and anti-CD33 Abs by flow cytometry and representative flow graphs of mobilized PB CD34+ HPCs from three donors are illustrated. *(B) *Flow cytometry analysis of hematopoietic progenitor and differentiation markers after HPC expansion in LSC culture for 9 days. The CD34+ HPCs of cord blood and adult PB were analyzed and presented.

To verify expression of the various growth factors in LSCs, RNAs harvested from LSCs were analyzed by semi-quantitative RT-PCR (Figure 1B and 1C). We confirmed that lentivector expression was stable even after 50 passages in these cell lines (data not shown). Highly enriched human CD34^+ ^HPCs derived from adult mobilized peripheral blood and BM expressed high level of hematopoietic progenitor marker CD133 and low level of CD33 (Figure 2A). This culture system supported HPC expansion for both healthy donors and cancer patients; for example, adult peripheral blood (PB) HPCs expanded in LSC-KFT63b to about one hundred-fold in two to three weeks (Figure 2A). Surface phenotype analysis indicated that the *ex vivo *expanded HPCs gradually lost progenitor markers (CD34, CD90, and CD133), which was accompanied by increased expression of myeloid differentiation markers CD38 and CD33 (Figure 2B). Similar results have been obtained with mouse BM Sca1^+^Lin^- ^(lineage-minus) HPCs (data not shown).

### Differentiation and expansion of DCPs toward meyloid DC-like phenotype

**Figure 3:**
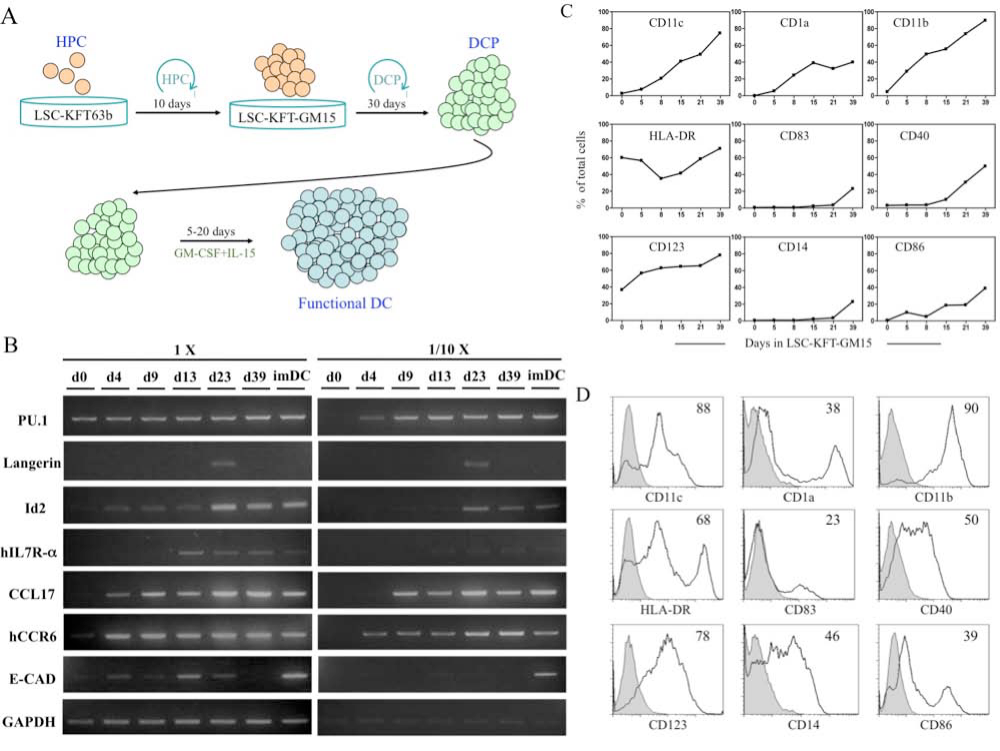
***Ex vivo *generation of functional DCs**. *(A) *Schematic illustration of expansion of human and mouse DCPs in culture. The human (CD34^+^) or mouse (Sca1^+^/Lin^-^) HPCs were cultured on LSC-KFT63b for 10 days, and then transferred to LSC-KFT-GM15 (LSC-KFT-mGM15 for mouse cells) to expand for 30 days. The resulting DCPs were further cultured in medium supplemented with GM-CSF, IL-15 and growth factors to induce immature and mature DCs. The average fold of expansion in each stage is indicated. *(B) *Kinetic analysis of myeloid cell differentiation markers. The expression kinetics of molecular markers for myeloid cells including PU.1, Langerin, Id2, hIL7R-α, CCL17, hCCR6, and E-cadherin (E-CAD) in the developing human DCPs were examined by RT-PCR. *(C) *Expression kinetics of DC surface markers of the DCPs based on flow cytometry analysis. *(D) *Surface phenotype of day 37 DCPs from the LSC-KFT-GM15 culture. The number inside each of the flow graph represents percentage of positive cells.

To see if the LSC culture system can generate functional DCs, we first expanded CD34^+ ^HPCs in LSC-KFT63b. After an initial 20-40-fold expansion, the cells were transferred to LSC-KFT-GM15. The DCPs continued to expand several orders of magnitude in 30 days; they were then transferred to feeder-free culture supplemented with GM-CSF and IL-15 to generate functional immature DCs as illustrated in Figure 3A.

Analysis of myeloid and DC lineage differentiation markers including PU.1, Langerin, Id2, hIL7R-a, CCL17, hCCR6, and E-cadherin (E-CAD) of the DCPs from day 0, 4, 9, 13, 23 and 39 by semi-quantitative RT-PCR revealed a gradual increase in myeloid (PU.1) and DC differentiation markers (Id2, hIL7R-a, CCL17, and hCCR6), and a stochastic expression of differentiating Langerhans cell markers (Langerin or CD207 and E-CAD) as compared with monocyte-derived immature DCs (imDC, Figure 3B). After 35 days, the DCPs displayed a differentiation profile similar to that of monocyte-derived imDCs, except for E-CAD, which was down-regulated. Kinetic analysis of monocyte and DC markers including CD14, CD11c, CD1a, CD11b, HLA-DR, CD83, CD40, CD123, and CD86 by flow cytometry showed that the *ex vivo*-expanded DCPs gradually differentiated toward mature DCs with increased expression kinetics of costimulatory molecules CD40, CD86, and DC maturation marker CD83 (Figure 3C). A representative flow cytometry analysis of DC markers of day 39 DCPs is shown in Figure 3D; at this time point, DCPs displayed increased activation and maturation markers resembling conventional mature DCs. Similar results were observed with mouse DCPs expanded in the LSC-KFT-mGM15 culture (not shown).

**Figure 4:**
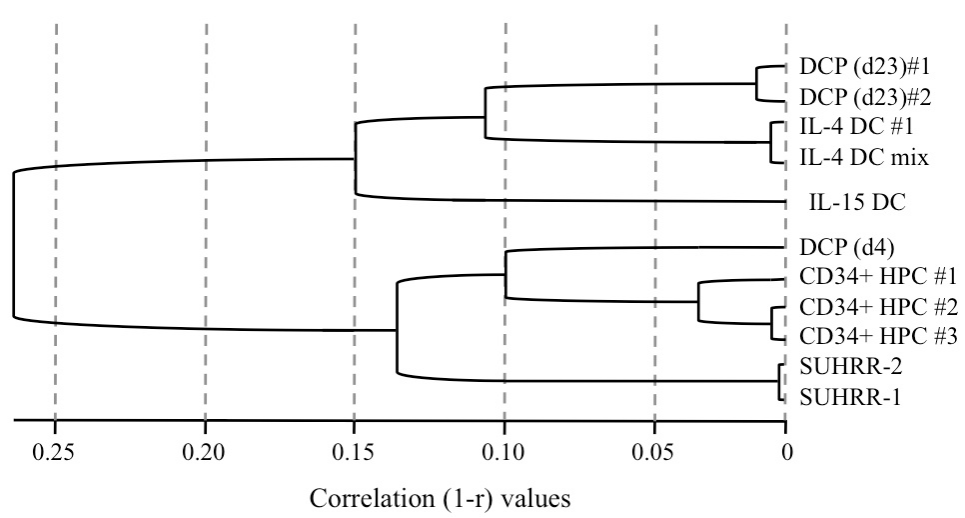
**Microarray dendogram analysis of *ex vivo *differentiated DCPs**. Gene expression profiles of CD34^+ ^HPC-derived DCPs at early (day 4) and late (day 23) time points after differentiation in LSC-KFT-GM15 were examined using Illumina human whole-genome RefSeq 8 expression BeadChip containing 24,000 genes. The related cell types are grouped in clusters in the dendogram, including day 4 and day 23 DCP, IL-4 DC (a single donor and a mix of five donors), IL-15 DC and three CD34+ HPC specimens. Stratagene Universal Human Reference RNA (SUHRR) was included for quality control.

We next examined the gene expression profile of the DCPs at different time points after differentiation from HPCs using Illumina BeadChip Human RefSeq-8 arrays. RNA samples were harvested from an early time point (day 4) and a late time point (day 23), and compared to RNAs harvested from CD34^+ ^HPCs and adherent PBMC-derived IL-4 and IL-15 DCs. Cluster analysis of unnormalized sample data showed that all biological replicates (two DCPs, two IL-4 DCs and three CD34^+ ^HPCs) sorted into the same groups (Figure 4). Sample dendogram revealed that the day 4 differentiated DCPs displayed gene expression profile resembled CD34^+^HPCs, whereas the day 23 differentiated DCPs displayed expression profile resembled IL-4 DCs (Figure 4).

### Cytokine and chemokine secretion profiles of the *ex vivo *generated DCs

As the morphology and surface marker expression pattern of the HPC-DCPs resembled myeloid DCs, we further examined the expression profile of inflammatory cytokines and chemokines. For comparison, we included the conventional IL-4 DCs and IL-15 DCs generated from adherent PBMCs. HPC-DCPs from adult BM CD34^+ ^HPCs were kept in feeder-free culture supplemented with GM-CSF and IL-15 to generate immature DCs and then were treated with TNF-α and LPS to induce maturation, and after extensive washes, the cells were incubated in serum-free AIM-V medium at a density of 1x10^6 ^cells per ml for 24 hr. The supernatants were collected and analyzed using a multiplex ELISA array, which simultaneously measures a panel of 23 cytokines and chemokines [35]. The results from two donors are summarized in Table 1. We noted that HPC-DCPs displayed a trend of upregulation of inflammatory cytokines and chemokines, with marked increase in IL-1b, IL-6, GRO-α (CXCL1), I-309 (CCL1), MCP-1 (CCL2), and MCP-2 (CCL8) compared with the traditional IL-4 DCs, suggesting that the DCPs have potent proinflammatory leukocyte chemotactic and activating functions. The overall cytokine and chemokine profile of DCP-derived DCs mimicked those of the IL-15 DCs except that the DCP-derived DCs produced reduced levels of IFN-γ and TNFα, at levels similar to those of the IL-4 DCs, yet with substantially increased expression of GRO-α and MCP-1.

**Table 1:** Analysis of cytokines and chemokines secreted by mature DCs

Cytokines/Chemokines	IL-4 DCs (pg/ml)	IL-15 DCs (pg/ml)	DCPs (pg/ml)
IL-1α	135 (32)	332 (1,228) ↑	1,202 (239) ↑
IL-1β	338 (92)	601 (2,696) ↑	8,582 (2,440) ↑↑
IL-2	6 (5)	7 (7)	6 (4)
IL-4	3 (3)	2 (4)	2 (1) ↓
IL-5	2 (1)	4 (7) ↑	9 (6) ↑
IL-6	652 (53)	1,664 (8,036) ↑	43,607 (4,605) ↑↑
IL-8	154,440 (55,237)	155,499 (297,615) ↑	303,222 (286,234) ↑
IL-10	68 (53)	7 (114)	110 (162) ↑
IL-12p70	6 (3)	16 (25) ↑	8 (6) ↑
IL-13	6 (5)	170 (148) ↑↑	10 (11) ↑
IL-15	25 (23)	2,359 (248) ↑↑	87 (81) ↑
IL-17	8 (4)	12 (13) ↑	5 (6)
IL-23	17 (30)	79 (90) ↑	215 (36) ↑
IFN-γ	< 1 ( < 1)	2,331 (1,905) ↑↑	< 1 (1)
TNFα	149 (72)	1,439 (3,152) ↑↑	226 (70)
TNFβ	< 1 (2)	87 (67) ↑	3 (6) ↑
Eotaxin	2 (3)	3 (3)	4 (2)
GRO-α	8,394 (2,158)	2,142 (15,744)	131,310 (19,241) ↑↑
I-309	1,989 (533)	15,086 (28,758) ↑	18,141 (42,805) ↑↑
MCP-1	342 (571)	547 (4,971) ↑	184,457 (127,001) ↑↑
MCP-2	11 (9)	339 (2,512) ↑↑	2,381 (46) ↑
RANTES	1,588 (179)	2,552 (1,875) ↑	1,666 (3,085) ↑
TARC	469,352 (337,109)	1,783 (1,636) ↓	20,739 (14,855) ↓

Results are averages of triplicate analyses of cytokines and chemokines (pg/ml/10^6 ^cells/24 hr) secreted by mature DCs of two donors (the 2^nd ^donor shown in parenthesis) using multiplex ELISA arrays. Up-(↑) and down-regulations (↓) are depicted by arrows, and double arrows indicate a difference more than ten-fold from the IL-4 DCs.

### Antigen capture and T cell activation functions of the *ex vivo *generated DCs

**Figure 5:**
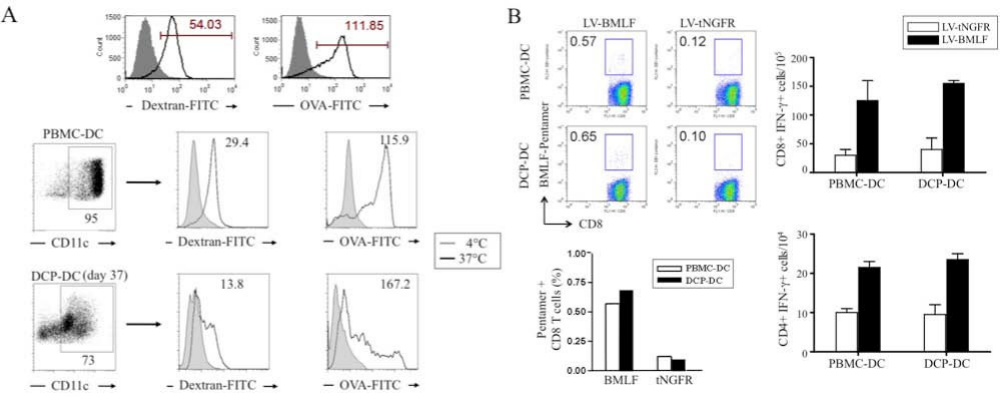
**Functional analyses of the *ex vivo *expanded DCPs**. *(A) *Analysis of antigen capture function of the *ex vivo *derived DCPs. Antigen capture was demonstrated using dextran-FITC or OVA-FITC particle internalization followed by flow cytometry analysis. Examples of FITC-positive control PBMC-derived DCs are shown at top; antigen capture was detected at 37°C but not at 4°C. *(B) *Analysis of antigen-specific T cell stimulation function. DCs were transduced with LVs encoding a control truncated NGFR (tNGFR) protein or BMLF protein of EBV, and incubated with autologous T cells (from the same HLA-A*0201 donor) for 10-12 days. The BMLF-specific A*0201 TCR bearing T cells were detected using a PE-conjugated MHC-peptide pentamer by flow cytometry (left panel). Antigen-specific effector function was analyzed based on intracellular expression of IFN-γ as described in Materials and Methods.

Professional antigen presenting cells can uptake and process antigens and stimulate T cells. To examine these functions, we compared the *ex vivo *generated DCs with immature adherent PBMC-derived DCs (PBMC-DCs) for their antigen uptake function by feeding them with fluorescent DQ-OVA (OVA-FITC) and dextran-FITC particles. The PBMC-DCs captured fluorescent particles at 37°C but not at 4°C, as demonstrated with flow cytometry. The day 37 DCP-DCs, which contained a large number of CD11c-positive cells, captured antigens as efficiently as did the PBMC-DCs (Figure 5A).

To see if the DCP-derived DCs were capable of activating antigen-specific T cells, we set up a DC/T cell coculture system as previously described [31,35,37]. The DCs were transduced with LVs encoding a viral antigen, EBV BMLF (LV-BMLF), or a truncated self-antigen tNGFR (LV-tNGFR). After maturation, the DCs were cocultured with autologous monocyte-depleted PBMCs for 12 days to generate antigen-specific T cells. The activated T cells were restimulated with the corresponding DCs and incubated with a BMLF peptide-specific (GLCTLVAML, HLA-A2*-restricted) MHC pentamer to detect antigen-specific response. Both the DCP-DCs and the PBMC-DCs induced antigen-specific T cell response when transduced with LV-BMLF, but not LV-tNGFR (Figure 5B, left panel). Intracellular staining for IFN-γ expression in CD4 and CD8 T cells confirmed that the DCP-DCs activated BMLF-specific T cells as effectively as did the PBMC-DCs (Figure 5B, right panel). In contrast, the self-antigen tNGFR did not register a substantial response.

### *In vivo *tumor suppression mediated by DCP-DCs derived from tumor-bearing mice

**Figure 6:**
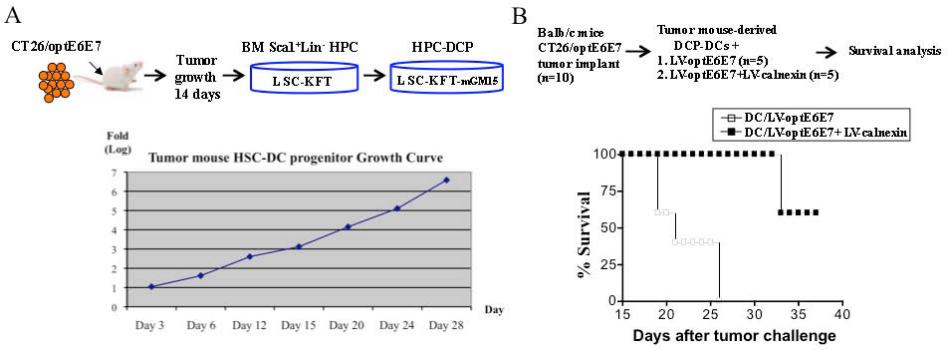
**DCPs derived from BM HPCs of tumor-bearing mice suppress tumor growth *in vivo***. *(A) Ex vivo *expansion of DCPs from BM HPCs of tumor-bearing Bulb/c mice. Balb/c mice were injected with CT26/optE6E7 tumor cells, and after 14 days, BM HPCs (Sca1^+^/Lin^-^) were harvested and cultured on LSC-KFT cells. After ten days, the expanded HPCs were transferred to LSC-KFT-mGM15 to generate DCPs. A representative mouse DCP expansion growth curve is shown. *(B) *Suppression of tumor growth in Balb/c mice immunized with LV-modified DCPs. CT27/optE6E7 tumor cells were first established in Balb/c mice. The mice were immunized with the *ex vivo *expanded DCPs, which were derived from the BM HPCs of CT26/optE67 tumor-bearing Balb/c mice. Two types of LV-modified DCs were tested: DCs transduced either by LV-optE6E7 alone or by LV-optE6E7 plus LV-calnexin. The percentages of survival of the two groups of mice are illustrated. Survival was based on tumor size smaller than 1 cm^3 ^without lesions.

The above assays demonstrated that the HPC-derived DCs displayed antigen presentation and T cell activation functions similar to that of monocyte-derived DCs. To examine their therapeutic potential, we designed an experiment using a previously established syngeneic mouse tumor model. BALB/c mice implanted with CT26 colon cancer cells expressing a codon-optimized Human Papilloma Virus 16 (HPV 16) E6 and E7 fusion protein (optE6E7) are protected through immunization with DCs transduced with LVs encoding optE6E7 and calnexin, a chaperone protein [31,35]. To mimic situation in a cancer patient, we derived DCPs from BM of tumor-bearing mice to see if a protective anti-cancer immune response can be induced. Figure 6A illustrates the strategy of generating DCPs from BM of tumor-bearing mice. CT26/optE6E7 tumors were first established in BALB/c mice, after confirming tumor growth, Sca1^+^Lin^- ^HPCs were purified from BM of the tumor-bearing mice. The HPCs were expanded in the LSC culture system as described; a representative mouse DCP expansion curve is illustrated (Figure 6A). The mouse DCPs expanded more than 6 orders of magnitude within 30 days. To generate DC vaccines, the tumor mouse-derived DCPs were differentiated for two days in IL-15 DC medium as described in Materials and Methods and transduced with LV-optE6E7 or LV-optE6E7 plus LV-calnexin. BALB/c mice bearing CT26/optE6E7 tumors were divided into two groups of five mice each group, and received two DC vaccinations at a one-week interval. We observed that mice injected with DCs modified by LV-optE6E7 plus LV-calnexin displayed increased survival than those modified by LV-optE6E7 alone (Figure 6B), a result consistent with previous findings using BM-derived IL-4 DCs [35].

## Discussion

The access to good quality and sufficient amount of functional DCs is critical to the success of immunotherapy against cancer and infections. In such settings, it is desirable to generate a large number of DCs capable of activating antigen-specific effector T cells but not T regulatory cells (Tregs). For examples, infusion of myeloid DCs and systemic administration of IL-2 have been shown to induce and expand CD4^+^FoxP3^+ ^Tregs in myeloma and renal cancer patients [31,40-42]. Here we present a reliable and highly reproducible strategy based on expansion of CD34^+ ^HPCs as well as differentiation of a unique lineage of functional DC progenitors (DCPs) using stromal cells engineered with lentivectors encoding multiple growth factors. The *ex vivo *generated DCs exhibited canonical antigen presentation functions including antigen uptake, processing and activation of T cells and *in vivo *anti-cancer effects.

The CD34^+ ^HPCs constitute a heterogeneous cell population that can generate various lineages of DCs [10,15,19,43,44]. We adopted a culture condition which supports expansion of CD34^+ ^HPCs and DC differentiation through the combination of cell-free and cell-associated signals including those supporting hematopoietic stem cell proliferation (KL, FL, TPO, IL-3, IL-6, bFGF), myeloid DC differentiation (GM-CSF), as well as IL-15 which is known to promote leukocyte survival and expansion. This unique *ex vivo *culture condition supports expansion of a novel lineage of DCs that are different from the conventional myeloid DCs. It is plausible that the continued renewal of differentiating DCPs in such a system was due to the lack of IL-4 and TNF-α, the two common growth factors used in many reported methods and known to induce DC maturation and block proliferation thus limiting their expansion [30]. This novel lineage of DCs is different from the commonly known IL-4 DCs or IL-15 DCs. Multiplex cytokine and chemokine array analysis suggests that DCPs resemble IL-15 DCs except that DCPs expressed less TNFα and IFN-γ, but much higher levels of inflammatory cytokines (IL-1a, IL-1b, and IL-6) associated with high levels of chemotactic factors (GRO-α and MCP-1) as compared with IL-15 DCs (Table 1). The beadchip microarray analysis of gene expression profile indicates that the *ex vivo *differentiated DCPs share a common DC progenitor but branched in between the peripheral blood adherent cell-derived IL-4 DCs and IL-15 DCs (Fig. 4). Functional analysis shows that DCP-DCs are fully capable of antigen presentation and stimulation of antigen-specific T cells. We conclude that the *ex vivo *derived DCP-DCs represent a unique lineage of DCs displaying phenotype and function between IL-4 DCs that have prominent adaptive immune functions, and IL-15 DCs that have prominent innate immune functions (manuscript in preparation). Further developmental study may elucidate the *in vivo *identity of the HPC-derived DCPs.

Several studies have reported that in addition to KL, FL and TPO, other growth factors including bFGF, bone morphogenetic protein 4 (BMP4), IL-3, IL-6 or stromal cell derived factor-1 (SDF-1 or CXCL12) can help increase CD34^+ ^HPC expansion and maintain their undifferentiated state [45-47]. Epigenetic modification using DNA methyltransferase (DNMT) inhibitor 5-azacytidine (5-aza) and/or histone acetylase inhibitor trichostatin A (TSA) can block differentiation of hematopoietic stem cells and moderately promote their expansion [48]. Supplementation of these factors in the LSC system, however, does not further increase HPC expansion potential (data not shown). Nevertheless, this *ex vivo *system offers a convenient and reproducible two-dimensional culture system for the study of self-renewal and development of human HSC and DC. Analysis of additional regulatory factors can be easily integrated into this culture system.

The development and maturation of DCs in cancer patients may be functionally defective, resulting in reduced expression of class II MHC and diminished antigen cross-priming activity [7,49-51]. In a previous report, we have shown that IL-4 DCs from multiple myeloma patients can be functionally improved through upregulation of the chaperone protein calnexin, which substantially increases the secretion of inflammatory cytokines and chemokines accompanied by a strong memory T cell response [31,35]; DCP-DCs, as illustrated here, may accomplish the same without further modifications. As IL-15 has been shown to reduce Treg activities and increase antigen-specific CD8 T cell response *in vitro *and *in vivo*, the *ex vivo *generated DCP-DCs have potential of overcoming DC dysfunctions in cancer patients [26,52,53]. DCPs from cancer patients including multiple myeloma, acute myeloid leukemia, acute lymphoblastic leukemia, Hodgkin's lymphoma and glioblastoma patients have been successfully generated from a small number of BM CD34^+ ^HPCs over several orders of magnitude ( > 10^6^) (unpublished). Thus, this *ex vivo *approach avoids potential immune suppressive microenvironment of DC development in patients. Further efforts in validated GMP process development and standardization of the feeder culture system are needed before DCP-DCs are ready for clinical trials.

## Conclusions

This *ex vivo *DC development system supports a robust expansion of a novel DC lineage in culture from a small number of CD34^+ ^HPCs, which provides a critical solution to problems often encountered in immunotherapy.

## List of abbreviations

BM: bone marrow; PB: peripheral blood; HPC: hematopoietic progenitor cell; PBM: peripheral blood monocyte; LSC: lentivector-modified stromal cell; LV: lentiviral vector; TPO: thrombopoietin; KL: kit-ligand; FL: Flt3-ligand; bFGF: basic fibroblast growth factor; DC: dendritic cell; DCP: DC progenitor; tNGFR: truncated nerve growth factor receptor; BLCL: B lymphoblastoid cell line; ICCS: intracellular cytokine staining.

## Competing interests

Yichen Wang was a paid employee of Vectorite Biomedica Inc. The University of Florida holds patents on modified dendritic cells and related inventions. Dr. Chang is entitled to a share of the sales royalty received by the University. The terms of these arrangements have been reviewed and approved by the University in accordance with its conflict of interest policies.

## Authors' contributions

All authors have read and approved the final manuscript, and are accountable for the integrity of the research and analysis of the data; LJC is responsible for the conception and execution; SH, YW, EP, SO and BW are responsible for data collection an analysis; LJC is responsible for initial drafting of the manuscript and all are responsible for revisions of the manuscript.

## Supplementary Material

Additional file 1 **Table S1**. Antibodies and their specific clones: Antibodies were purchased from BD PharMingen, Invitrogen CALTAG laboratories, eBiosciences and Cell Signaling. Click here for file

Additional file 2 **Table S2**. Primer sequences for cDNA cloning and RT-PCR. Click here for file
