# Supplementary material for: Ex vivo development, expansion and in vivo analysis of a novel lineage of dendritic cells from hematopoietic stem cells
Source: J Immune Based Ther Vaccines. 2010 Nov 24;8:8. doi: 10.1186/1476-8518-8-8 (PMC3004889; doi:10.1186/1476-8518-8-8)
Supplement: Additional file 1 — Table S1. Antibodies and their specific clones: Antibodies were purchased from BD PharMingen, Invitrogen CALTAG laboratories, eBiosciences and Cell Signaling. [file 1476-8518-8-8-S1.DOC]

# Additional file

Table S1. Antibodies and their specific clones: All antibodies were purchased from BD PharMingen, Invitrogen CALTAG laboratories, eBiosciences and Cell Signaling.

| Antibody | Clone | Label | Source |
| --- | --- | --- | --- |
| CD1a | HI149 | APC | BD Pharmingen |
| CD3 | SK7  S4.1  UCHT1  S4.1  HIT3a  S4.1  HIT3a  UCHT1  UCHT1  SK7  UCHT1 | PE-Cy7  PE  PE-Cy5  FITC  FITC  APC  APC  Alexa Fluor 647  Alexa Fluor 488  AmCyan  Pacific blue | BD Pharmingen  Invitrogen CALTAG  BD Pharmingen  Invitrogen CALTAG  BD Pharmingen  Invitrogen CALTAG  BD Pharmingen  BD Pharmingen  BD Pharmingen  BD Pharmingen  BD Pharmingen |
| CD4 | RPA-T4  S3.5  RPA-T4  SK3  RPA-T4  ---  SK3  RPA-T4  S3.5 | APC  FITC  FITC  PE  PE  PE-Cy7  PE-Cy7  Pacific blue  PE-TR | BD Pharmingen  Invitrogen CALTAG  BD Pharmingen  BD Pharmingen  BD Pharmingen  Invitrogen CALTAG  BD Pharmingen  BD Pharmingen  Invitrogen CALTAG |
| CD8 | RPA-T8  ---  RPA-T8  SK1  SK1  SK1  RPA-T8  RPA-T8  3B5 | PE  FITC  FITC  PerCP  PerCP—Cy5.5  APC-Cy7  PE-Cy7  Pacific blue  Alexa 700 | BD Pharmingen  Invitrogen CALTAG  BD Pharmingen  BD Pharmingen  BD Pharmingen  BD Pharmingen  BD Pharmingen  BD Pharmingen  Invitrogen CALTAG |
| CD11b | D12 | PE, APC | BD Pharmingen |
| CD11c | BU15  PE  PE-Cy7  APC  APC  PE-Cy5 | FITC  B-ly6  3.9  BU15  B-ly6  B-ly6 | Invitrogen CALTAG  BD Pharmingen  eBiosciences  Invitrogen CALTAG  BD Pharmingen  BD Pharmingen |
| CD14 | MP9  M5E2  Tuk 4  Tuk 4  M5E2 | PE, PerCP  APC  PE  FITC  Pacific blue | BD Pharmingen  BD Pharmingen  Invitrogen CALTAG  Invitrogen CALTAG  BD Pharmingen |
| CD19 | SJ25-C1  SJ25-C1  HIB19  SJ25-C1 | PE  FITC  FITC  Amcyan | Invitrogen CALTAG  BD Pharmingen  BD Pharmingen  BD Pharmingen |
| CD33 | CD43-4D3  P67.6  WM53 | APC  PerCP-Cy5.5  PE-Cy5 | Invitrogen CALTAG BD Pharmingen  BD Pharmingen |
| CD34 | 563  581 (Class III)  8G12  AC136  8G12 | PE  FITC  PE  PE  PE-Cy7 | BD Pharmingen  Invitrogen CALTAG  BD Pharmingen  Miltenyi Biotec  BD Pharmingen |
| CD69 | F950  L78 | APC  FITC | BD Pharmingen  BD Pharmingen |
| CD80 | L307.4 | Cy-C | BD Pharmingen |
| CD83 | HB15e  HB15e  HB15e | Purified  PE  APC | BD Pharmingen  BD Pharmingen  Invitrogen CALTAG |
| CD86 | BU63 | R-PE | Invitrogen CALTAG |
| CD90 | 5E10  5E10  5E10  5E10 | Purified  PE  PE-Cy5  APC | BD Pharmingen  BD Pharmingen  BD Pharmingen  BD Pharmingen |
| CD123 | 7G3  7G3 | PE  PE-Cy5 | BD Pharmingen  BD Pharmingen |
| CD133 | AC133 | APC | Miltenyi Biotec |
| HLA-ABC | G46-2.6 | FITC | BD Pharmingen |
| HLA-DR | TU36  TU36 | FITC  PE | Invitrogen CALTAG  Invitrogen CALTAG |
| IFN-γ | 25723.11  25723.11  B27 | Alexa Fluor 700  APC  FITC | BD Pharmingen  BD Pharmingen  BD Pharmingen |
| TNFα | MP9-20A4  MAb11 | APC  APC | Invitrogen CALTAG  BD Pharmingen |
|  | MAb11 | PE | BD Pharmingen |
|  |  |  |  |
