# Supplementary material for: Ex vivo development, expansion and in vivo analysis of a novel lineage of dendritic cells from hematopoietic stem cells
Source: J Immune Based Ther Vaccines. 2010 Nov 24;8:8. doi: 10.1186/1476-8518-8-8 (PMC3004889; doi:10.1186/1476-8518-8-8)
Supplement: Additional file 2 — Table S2. Primer sequences for cDNA cloning and RT-PCR. [file 1476-8518-8-8-S2.DOC]

# Additional File

**Table S2**. Primer sequences for cDNA cloning and RT-PCR.

Primer name Primer sequence (5’ to 3’)

hIL-3 RT-PCR F TGATCGACGAGATCATCACC

hIL-3 RT-PCR R GCAGGTTCTTCAGGATGCTC

hIL-6 ORF F AAGGATCCACCATGAACTCCTTCTCCACAAGC

hIL-6 ORF R AAACTAGTCTACATTTGCCGAAGAGCC

hIL-6 RT-PCR F GTAGCCGCCCCACACAGACAGCC

hIL-6 RT-PCR R GCCATCTTTGGAAGGTTCAGG

hIL-15 ORF F TTGGATCCACCATGAGAATTTCGAAACCACATTTG

hIL-15 ORF R TTACTAGTCAAGAAGTGTTGATGAAC

hIL-15 RT-PCR F AGCTGGCATTCATGTCTTCA

hIL-15 RT-PCR R ACTTTGCAACTGGGGTGAAC

hGM-CSF ORF F CCCGGGAAGCTTCCACCATGTGGCTGCAGAGCCTG

hGM-CSF ORF R AATGGATCCTATCACTCCTGGACTGGCTC

hGM-CSF RT-PCR F ATGTGAATGCCATCCAGGAG

hGM-CSF RT-PCR R AGGGCAGTGCTGCTTGTAGT

mGM-CSF ORF F aat cta gac cac cat gtg gct gca gaa ttt ac

mGM-CSF ORF R aagaattcctcatttttggactgg

mGM-CSF RT-PCR F GGCCTTGGAAGCATGTAGAG

mGM-CSF RT-PCR R CCGTAGACCCTGCTCGAATA

hbFGF ORF F AAGGATCCACCATGGTGGGTGTCGGGGGTGGAG

hbFGF ORF R AAACTAGTCAGCTCTTAGCAGACATTG

hbFGF RT-PCR F ATGGCAGCCGGGAGCATCACCACGC

hbFGF RT-PCR R CAGCTCTTAGCAGACATTGGAAGAAAAAG

hSCF ORF F TTTCTAGACCACCATGAAGAAGACACAAACTTG

hSCF ORF R CCGGATCCTTACACTTCTTGAAACTC

hSCF RT-PCR F CTCCTATTTAATCCTCTCGTC

hSCF RT-PCR R TACTACCATCTCGCTTATCCA

hFlt3-L ORF F aaggatccgcaggatgaggccttg

hFlt3-L ORF R cccaggatgaggccttgg

hFlt3-L RT-PCR F GCT TCA AGA TTA CCC AGT CAC C

hFlt3-L RT-PCR R GAC CCA GCG ACA GTC TTG A

hTPO ORF F TTTCTAGACCACCATGGAGCTGACTGAATTG

hTPO ORF R TTGAATTCTTACCCTTCCTGAGACAG

hTPO RT-PCR F GAA TGG AAA ACC CAG ATG GA

hTPO RT-PCR R AGG GAT GAG AGG CAA GTG G

EBV BMLF ORF F AAGGATCCACCATGGAGGGCAGCGAAGAACAC

EBV BMLF ORF R AAA CTA GTT ATT GAT TTA ATC CAG GAA C

hCCL17 RT-PCR F ATG GCC CCA CTG AAG ATG CTT

hCCL17 RT-PCR R TGA ACA CCA ACG GTG GAG G

hPU.1 RT-PCR F TGG AAG GGT TTC CCC TCG TC

hPU.1 RT-PCR R TGC TGT CCT TCA TGT CGC CG

hCCR6 RT-PCR F GGGGGAATATTCTGGTGGTGA

hCCR6 RT-PCR R CATCGCTGCCTTGGGTGTTGTAT

hE-CAD RT-PCR F TCTACAGCATCACTGCCCAAGGAGCTG

hE-CAD RT-PCR R AGCTTGAACCACCAGGGTATACGTAGG

hLangerine RT-PCR F GCTTGGAGAATATGAGCAAGTTGC

hLangerine RT-PCR R GCACTTTGGACCTTGTTGAATGGC

hId2 RT-PCR F ACGACCCGATGAGCCTGCTA

hId2 RT-PCR R TCCTGGAGCGCTGGTTCTG

hIL7Ra RT-PCR F ATTCAAGCTAGAGATGAAGTG

hIL7Ra RT-PCR F TTACTCTTTCATTCTTTCCTC

PreT RT-PCR F AGT ACA CAG CCC ATG CAT CTG TCA

PreT RT-PCR R AAT GCT CCA AGA CTG GAG GAA GGA

mGAPDH RT-PCR F TCACCACCATGGAGAAGGC

mGAPDH RT-PCR R GCTAAGCAGTTGGTGGTGCA

ORF, open reading frame; F, forward; R, reverse.
